# Infectious Causes of Stillbirths: A Descriptive Etiological Study in Uganda

**DOI:** 10.1093/ofid/ofae606

**Published:** 2025-03-10

**Authors:** Lauren Hookham, Valerie Tusubira, Amusa Wamawobe, Dan R Shelley, Caitlin Farley, Edward A R Portal, Simon Beach, Hannah G Davies, Konstantinos Karampatsas, Mary Kyohere, Joseph Peacock, Philippa Musoke, Owen B Spiller, Paul T Heath, Musa Sekikubo, Kirsty Le Doare, PROGRESS Study Author Group, Abdelmajid Djennad, Agnes Nyamaizi, Agnes Ssali, Alexander Amone, Amusa Wamawobe, Annettee Nakimuli, Caitlin Farley, Carol Nanyunja, Christine Najuka, Cleophas Komugisha, Dan R Shelley, Edward A R Portal, Ellie Duckworth, Emilie Karafillakis, Geraldine O’Hara, Godfrey Matovu, Hannah G Davies, Janet Seeley, Joseph Peacock, Juliet Nsimire Sendagala, Katie Cowie, Kirsty Le Doare, Konstantinos Karampatsas, Lauren Hookham, Madeleine Cochet, Margaret Sewegaba, Mary Kyohere, Maxensia Owor, Melanie Etti, Merryn Voysey, Moses Musooko, Musa Sekikubo, Owen B Spiller, Patience Atuhaire, Paul T Heath, Philippa Musoke, Phiona Nalubega, Pooja Ravji, Richard Katungye, Ritah Namugumya, Rosalin Parks, Rose Azuba, Sam Kipyeko, Simon Beach, Stephen Bentley, Tim Old, Tobius Mutabazi, Valerie Tusubira, Vicki Chalker

**Affiliations:** Institute for Infection and Immunity, St George's University of London, London, UK; Makerere University—Johns Hopkins University (MUJHU) Research Collaboration, Kampala, Uganda; Medical Research Council/Uganda Virus Research Institute and London School of Hygiene & Tropical Medicine Uganda Research Unit, Entebbe, Uganda; Makerere University—Johns Hopkins University (MUJHU) Research Collaboration, Kampala, Uganda; Department of Medical Microbiology, Makerere University, Kampala, Uganda; Division of Infection & Immunity, Cardiff University School of Medicine, Cardiff, UK; Division of Infection & Immunity, Cardiff University School of Medicine, Cardiff, UK; Division of Infection & Immunity, Cardiff University School of Medicine, Cardiff, UK; Department of Obstetrics and Gynaecology, Makerere University, Kampala, Uganda; Institute for Infection and Immunity, St George's University of London, London, UK; Institute for Infection and Immunity, St George's University of London, London, UK; Makerere University—Johns Hopkins University (MUJHU) Research Collaboration, Kampala, Uganda; Department of Clinical Research, Faculty of Infectious and Tropical Diseases, London School of Hygiene & Tropical Medicine, London; Institute for Infection and Immunity, St George's University of London, London, UK; Institute for Infection and Immunity, St George's University of London, London, UK; Makerere University—Johns Hopkins University (MUJHU) Research Collaboration, Kampala, Uganda; Institute for Infection and Immunity, St George's University of London, London, UK; Makerere University—Johns Hopkins University (MUJHU) Research Collaboration, Kampala, Uganda; Division of Infection & Immunity, Cardiff University School of Medicine, Cardiff, UK; Institute for Infection and Immunity, St George's, University of London, London, UK; Department of Obstetrics and Gynaecology, Makerere University, Kampala, Uganda; Institute for Infection and Immunity, St George's University of London, London, UK; Makerere University—Johns Hopkins University (MUJHU) Research Collaboration, Kampala, Uganda; Medical Research Council/Uganda Virus Research Institute and London School of Hygiene & Tropical Medicine Uganda Research Unit, Entebbe, Uganda

**Keywords:** antimicrobial resistance, infection, pregnancy, sepsis, stillbirth

## Abstract

**Background:**

Every year an estimated 2–3 million babies are stillborn, with a high burden in Africa. Infection is an important driver of stillbirth. There is a lack of data on the bacterial causes of stillbirth in Uganda, contributing to a lack of interventions such as effective prophylaxis and development of maternal vaccine options against the most implicated pathogens.

**Methods:**

The PROGRESS study was an observational cohort study undertaken in Kampala, Uganda, between November 2018 and April 2021. If a woman delivered a stillborn baby, consent was sought for the collection of a heart-blood aspirate. One to three mL of blood was collected and sent for culture using the BD Bactec blood culture system. Organism identification was performed using biochemical testing and matrix-assisted laser desorption/ionization–time of flight mass spectrometry. Susceptibilities to appropriate panels of antimicrobials were determined by agar dilution.

**Results:**

Kawempe Hospital registered 34 517 births in the study period, of which 1717 (5.0%) were stillbirths. A total of 581 (33.8%) were recruited into the study, and heart blood aspirates were performed on 569 (97.9%). Blood samples were sufficient for analysis of 476, with a total of 108 positive cultures (22.7% of sampled stillbirths). Fifty-nine of 108 blood cultures contained organisms that were considered potential pathogens, giving a pathogen positivity rate of 12.4%. Common pathogens included *Enterococcus* spp. (n = 14), *Escherichia coli* (n = 13), viridans streptococci (n = 18), *Klebsiella pneumoniae* (n = 6), and group B *Streptococcus* (n = 5). Gram-negative organisms were frequently resistant to commonly used first-line antimicrobials.

**Conclusions:**

The high proportion of stillbirths caused by likely pathogenic bacteria in Uganda highlights the potential for prevention with prophylaxis and stresses the need for further investment in this area.

The global burden of stillbirth, a baby born with no signs of life after a given threshold [1], is focused in Sub-Saharan Africa and Southern Asia, where 3 out of 4 stillbirths occur [2]. Within Sub-Saharan Africa, rates have been reported of up to 32.2 per 1000 births [3], yet this could still be underreported due to limitations within health care systems [4]. In Uganda, the stillbirth rate has been reported nationally as 17.8 per 1000 births [5], but this varies by district and region, with estimates of up to 20 per 1000 births in Northern Uganda, with infection listed as the probable cause in 35% of cases [6]. Though maternal health targets were a feature of the Millennium Development Goals [7] and of the 2030 Agenda for Sustainable Development [8], targets to tackle stillbirth are still lacking.

Stillbirths may occur for a variety of reasons and at different time points of pregnancy and delivery. Stillbirths may be categorized by presumed etiology and may include maternal, placental, and fetal factors. Maternal or fetal infection is thought to cause between 10% and 25% of stillbirths, with the majority thought to be related to ascending bacterial infection [9]. Infection may cause stillbirth through a variety of mechanisms, which may include placental damage, direct fetal infection, and severe maternal infection [10] that does not require transmission of pathogen to placenta or fetus. Bacterial infection may be confirmed by evidence of pathogens in postpartum specimens such as blood cultures. Critically, infection is a potentially preventable cause of stillbirth if appropriate antibiotic prophylaxis or treatment can be identified. The development of vaccines against common pathogens such as group B *Streptococcus* (GBS) or *Escherichia coli* (*E. coli*) could reduce the burden of stillbirths globally, but burden data are lacking to inform vaccine efficacy estimates against stillbirths.

This paper forms part of a supplement based on the PROGRESS study. The seroepidemiology of maternally derived antibodies against GBS in Mulago/Kawempe Hospitals Uganda (PROGRESS) study aimed to describe the causes of infectious mortality and morbidity as well as the seroepidemiology of GBS infection—the major cause of neonatal sepsis worldwide—in Kampala, Uganda [11]. This was a prospective cohort and nested case–control study, including a stillbirth surveillance arm. The culture of heart blood aspirates in this setting allows for analysis and discussion of the bacterial etiology of stillbirth in Uganda.

## METHODS

### Study Design and Methods

#### Study Design

The PROGRESS study was an observational study, and detailed information regarding the PROGRESS research protocol and overall validation results has been published separately [10].

We assessed the incidence of bloodstream infections in stillbirth and the key bacterial pathogens in a prospectively enrolled cohort. Participant recruitment sites for the studies that form part of this supplement are detailed in a flowchart available in the supplementary material of another paper published in this issue [12].

#### Study Setting

The study was based at Kawempe National Referral Hospital (KNRH), Kampala. This is the largest national referral hospital for pregnancies in Kampala, Uganda's capital city. KNRH is the referral facility for high-risk pregnancies from across the surrounding areas as well as serving the local community for routine antenatal and maternal care. There are an estimated 25 000 births per year at Kawempe.

The blood culture samples were processed at the Makerere University Clinical Microbiology Laboratory (MUCML), accredited by the College of American Pathologists. Confirmatory bacterial identification was performed at St George's, University of London, and antimicrobial susceptibility testing was performed at Cardiff University, United Kingdom.

#### Inclusion and Exclusion Criteria

Eligible participants included women over the age of 18 and emancipated minors between 14 and 17 years of age delivering a stillborn baby at Kawempe National Referral Hospital during the study period. Women were ineligible for the study if they were unable to give written informed consent. There are known challenges in defining stillbirth, with a variety of definitions in use across the medical literature [1]. Gestational age thresholds range between 20 and 28 weeks depending on health care and infrastructure across countries [13]. We defined stillbirth as a fetal death occurring before 28 weeks of gestation, in keeping with local guidelines. Gestational age was estimated using last menstrual period, fundal height, and, if available, ultrasound [14]. Fetal death was confirmed by the absence of signs of life at delivery, and both intrapartum and antepartum fetal deaths were included in this study.

#### Clinical Sampling

Collection of heart blood aspirates for culture began on December 4, 2019, and continued until December 31, 2021. Eligible participants were approached by study staff, and brief information about the study was given in the participant's preferred language (English or Luganda); verbal consent was taken to allow the collection of time-sensitive cord blood samples at delivery. If the woman delivered a stillborn baby, then consent was sought for collection of 5–10 mL of blood, aspirated via the heart. The heart blood aspirate sample was aliquoted, half the sample was sent for serological analysis (results awaiting publication separately), and 1–3 mL was sent for blood culture. In the case that the sample obtained was insufficient for both samples, the serological sample was prioritized. The volume of blood taken was documented on the sample request form. Blood was inoculated into a BD Bactec pediatric plus blood bottle [15] and then sent for culture at MUCML.

### Lab Methodology

#### Blood Culture Processing

All blood cultures were incubated in an automatic BACTEC machine (BACTEC 9050,9120 or FX40) [16]. Samples that arrived at the laboratory after 12 hours of collection and those that showed a physical turbidity on arrival were assessed and subcultured before loading into the blood culture machine. All BACTEC-positive cultures were gram-stained and subcultured onto MacConkey, 5% sheep blood (BA), and 5% chocolate blood agar (CBA; Biolab Hungary [17] and Oxoid UK [18]). MacConkey was incubated in ambient air, and BA and CBA in 5% carbon dioxide at 37°C. Samples that showed yeast cells or fungal elements in the gram reaction were additionally subcultured on Sabouraud's Chloramphenicol agar (SAB). Plates were monitored after every 24 hours for any growth. Blood culture bottles were incubated for 5 days, at which point they were deemed negative.

#### Identification of Organisms

Bacterial identification was based on morphology, gram stain, and standard biochemical tests for gram-positive and gram-negative identification. In the case of *Streptococcus* species, Lancefield Streptococcal Grouping Kit was used as part of the panel for identification [19]. Bacteria were then defined as likely pathogens or likely contaminants based upon the expertise of a panel of microbiologists and infectious disease physicians.

Positive blood cultures with an identified pathogen were shipped to St George's, University of London, and organism identification confirmed with matrix-assisted laser desorption ionization–time of flight mass spectrometry (MALDI-TOF MS) via rapid direct plating, as per the methodology in previous validation work [20]. If there was a discrepancy between different identification methods, the organism identified via MALDI-TOF was used.

#### Antimicrobial Susceptibility Testing

Antimicrobial susceptibility testing was performed at Cardiff University. Single colonies from overnight culture on Colombia blood agar of frozen archived isolates were taken and resuspended to prepare 0.5 McFarland standards in sterile 3-mL 0.85% saline (Oxoid, UK) for antimicrobial sensitivity testing. Minimum inhibitory concentrations (MICs) were determined for gram-positive (benzylpenicillin, chloramphenicol, clindamycin, erythromycin, gentamicin, levofloxacin, tetracycline, and vancomycin) and gram-negative bacteria (amikacin, amoxicillin-clavulanate, ampicillin, azithromycin, ceftazidime, chloramphenicol, ciprofloxacin, colistin, gentamicin, meropenem, tetracycline, and tigicycline) separately. Plates were inoculated using a multipoint inoculator (MAST URI DOT) post–autoclave sterilization of the pins. Inoculated plates were incubated at 37°C for 18–24 hours. Susceptibility was determined according to the European Committee on Antimicrobial Susceptibility Testing (EUCAST) guidelines [21] unless unavailable. In this case, Epidemiological Cutoff Values (ECOFF) or EUCAST-determined pharmacokinetic/pharmacodynamic values were used [21].

#### Data Management

Relevant clinical information was extracted from the mother's hand-held or hospital notes onto the case report form (CRF) in Research Electronic Data Capture (REDCap) [22], hosted at Makerere University Johns Hopkins University (MUJHU). This included the mother's demographic details, past medical, surgical, and obstetric history data, HIV status, infant gestation at birth (weeks, determined by Ballard score), last menstrual period (LMP), premature rupture of membranes (PROM), ultrasound date, receipt of intrapartum antibiotic prophylaxis (IAP), type of IAP, time between administration of first IAP and delivery, and mode of delivery. The timing of fetal death (intrapartum or antepartum) was also recorded as per clinical assessment.

#### Sample Size

There was no formal sample size calculation as this was a prospective study conducted within a larger cohort study, intended to assess the feasibility of recruiting and following 35 000 women and their infants and to ascertain the seroepidemiology of maternally derived antibody against GBS [11].

#### Statistical Analysis

For the main end point of this study, we have undertaken descriptive analyses. We report medians and interquartile ranges or means and standard deviations based on the distributions of the data. An analysis of maternal and infant factors associated with infectious stillbirths (compared with those where no pathogens were identified) was undertaken, and the chi-square test was performed to test the strength of these associations. Extensive efforts were undertaken to identify missing data. All missing data are reported in the included tables. We used STATA (version 17.1) for statistical analyses.

## RESULTS

**Figure 1.**
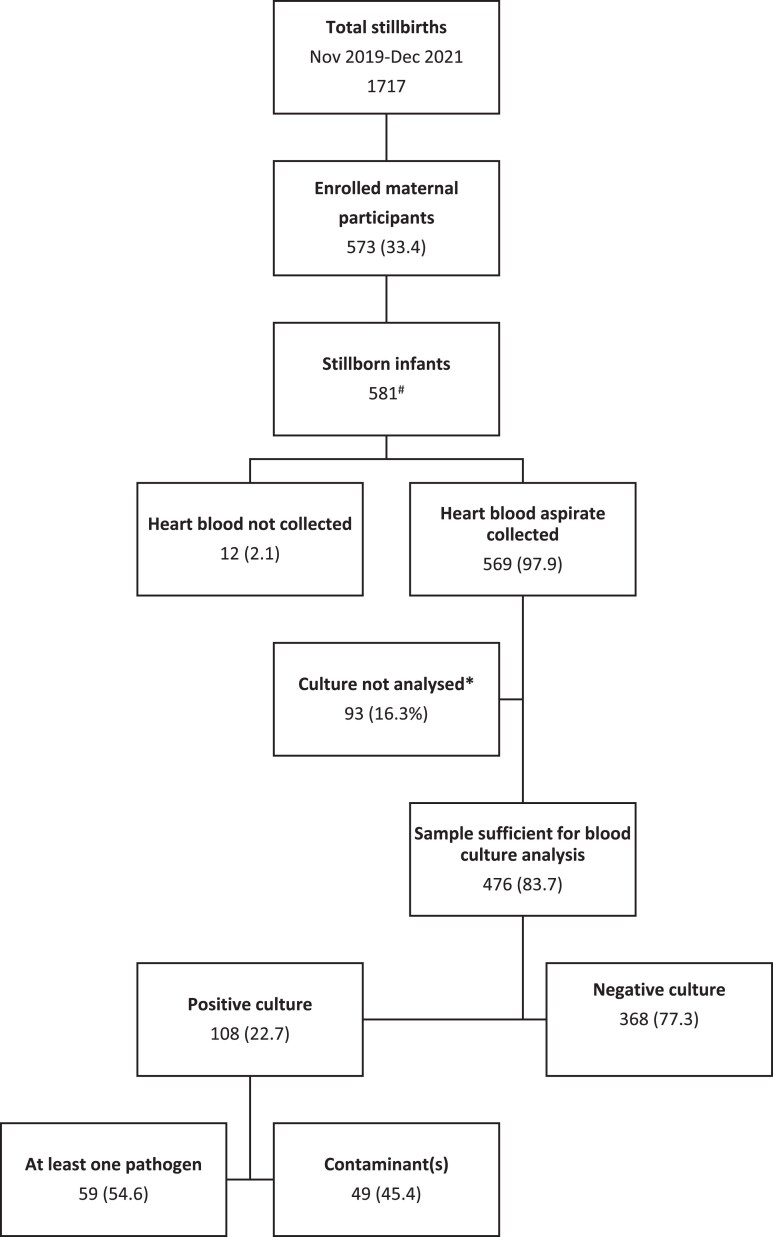
Flowchart for enrollment and sample collection for participants who delivered stillborn infants. ^a^Eight twin pregnancies.

Kawempe Hospital registered 34 517 births in the study period, of which 1717 (5.0%) were stillbirths. Five hundred eighty-one stillborn infants were recruited into the study (33.8%), with heart blood aspirates performed on 97.9% (n = 569). Samples were sufficient for analysis of 476 (84%) blood culture samples (Figure 1). The mean age of enrolled maternal participants (SD) was 26.9 (6.1) years. Forty-seven women (47/427 11.0%) had experienced a previous stillbirth. A small number of women (15/573 2.6%) were malnourished according to mid-upper arm circumference, and 128 (22.3%) were overweight or obese. Seventy-nine (13.8%) women were living with HIV, and 38 (6.6%) had been diagnosed with syphilis during pregnancy. Other maternal characteristics including complications of pregnancy and labor are described in Table 1. The majority of stillbirths were antepartum deaths (449/581, 77.3%). Over a third (36.1%) of stillbirths occurred preterm (Table 2).

**Table 1.** Demographics and Characteristics for Enrolled Maternal Participants

Enrolled Participants	n = 573
Maternal age	
Mean (SD), y	26.9 (6.1)
Maternal education level (n = 572)	
None	17 (3.0)
Primary education	177 (30.9)
Secondary	313 (54.7)
Tertiary/university	65 (11.4)
Parity (n = 573)	
Primigravida	146 (25.5)
Multigravida	427 (74.5)
Previous stillbirth (n = 427)	
Yes	47 (11.0)
No	380 (89.0)
Previous spontaneous abortion (425)	
Yes	125 (29.4)
No	300 (70.6)
Mid-upper arm circumference (n = 573)	
Median (IQR), cm	28 (26–30)
<23** **cm	15 (2.6)
23.0–30.0** **cm	430 (75.0)
>30** **cm	128 (22.3)
Maternal syphilis infection (n = 573)	
Positive	38 (6.6)
Negative	334 (58.2)
Not tested	201 (35.1)
Maternal participants with HIV (n = 568)	
HIV positive	79 (13.8)
HIV negative	489 (85.3)
Maternal complications**—**antenatal care (n = 573)	
Hypertensive disorder of pregnancy	118 (20.6)
Gestational diabetes	2 (0.4)
Malaria in pregnancy	35 (6.1)
Urinary tract infection	56 (9.6)
Anemia	13 (2.3)
Oligohydramnios	17 (3.0)
Polyhydramnios	8 (1.4)
Antepartum bleeding	46 (8.0)
Intrauterine growth restriction	3 (0.5)
Maternal complications of delivery (573)	
Dysfunctional labor	41 (7.2)
Suspected chorioamnionitis	5 (0.9)
Prolonged rupture of membranes	6 (1.0)
Placental abruption/previa	47 (8.2)
Cord prolapse	24 (4.2)

Abbreviation: IQR, interquartile range.

**Table 2.** Characteristics of Enrolled Stillborn Infants

Infant Characteristics	n = 581^a^
Sex (n = 581)	
Male	303 (52.2)
Female	275 (47.3)
Indeterminate	3 (0.5)
Birthweight grams (n = 579)	
Mean birthweight (SD)	2422 (954.8)
Normal birthweight	288 (49.6)
Low birthweight	184 (31.7)
Very low birthweight	109 (18.8)
Gestational age at birth (n = 579)	
Term	370 (63.9)
Preterm	209 (36.1)
Delivery presentation (n = 580)	
Cephalic	509 (87.8)
Breech	66 (11.4)
Transverse	5 (0.9)
Type of stillbirth (n = 581)	
Intrapartum	132 (22.7)
Antepartum	449 (77.3)

^a^Five hundred sixty-four singletons and 17 twins (8 twins where both babies were stillborn and 9 twins where 1 infant was liveborn and the second stillborn).

There were a total of 108 positive cultures, with a small subset (n = 4) having mixed growth (Table 3). Positive cultures were further categorized as likely pathogens or as likely contaminants using expert consensus. Following categorization, 59 heart blood aspirate samples were positive with at least 1 pathogen (Table 3; Supplementary Tables 1 and 2). This represents a blood culture yield of 12.4%. The majority of organisms were gram positive (40/63, 63.5%). Coagulase-negative staphylococci (21/50, 42.0%) were the most frequently identified contaminants (Supplementary Table 3).

**Table 3.** Pathogenic Organisms Identified in 59 Blood Cultures

Pathogens Identified (n = 63)^a^	Freq (%)
Gram negative	23 (36.5)
*Escherichia coli*	13 (20.6)
*Klebsiella pneumoniae*	6 (26.1)
Other Enterobacteraciae group organisms	2 (3.2)
Other gram-negative organisms	2 (3.2)
Gram positive	40 (63.5)
Viridans group *Streptococcus*	18 (45.0)
*Enterococcus* spp.	14 (35.0)
*Streptococcus agalactiae*	5 (12.5)
*Staphylococcus aureus*	3 (7.5)
Total	63 (100)

^a^Fifty-nine blood cultures were monomicrobial and 4 polymicrobial.

The most common pathogens identified by group were viridans streptococci (n = 18), *Enterococcus* spp. (n = 14), *Escherichia coli* (n = 13), *Klebsiella pneumoniae* (n = 6), and group B *Streptococcus* (n = 5) (Table 3). Heart blood aspirates positive with a pathogen were more frequent in infants of low birthweight (LBW; <2500 g). There was no difference between infants exposed to HIV in utero and those who were not, and there was no difference in maternal characteristics between those with positive and negative culture results (Table 4).

**Table 4.** Factors Associated With a Culture-Positive, Pathogen-Positive Stillbirth

	Non-Infection-Associated Stillbirth	Infection-Associated Stillbirth	
	(n = 413)	(n = 57)	*P* Value*
	Freq (row %)	Freq (row %)	
Maternal age			.19
15–25 y	182 (85.1)	32 (15.0)	
26–35 y	196 (90.7)	20 (9.3)	
>35 y	35 (87.5)	5 (12.5)	
Previous stillbirth			.80
No	271 (88.6)	35 (11.4)	
Yes	34 (87.2)	5 (12.8)	
Maternal MUAC			.77
<23** **cm (underweight)	9 (81.8)	2 (18.2)	
23–30** **cm (normal weight)	307 (87.7)	43 (12.3)	
>30** **cm (overweight/obese)	97 (89.0)	12 (11.0)	
Maternal education level			.10
None/primary	9 (90.0)	1 (10.0)	
Secondary	348 (86.6)	54 (13.4)	
Tertiary/university	55 (96.5)	2 (3.5)	
Maturity			.22
Term	266 (89.3)	32 (10.7)	
Preterm	146 (85.4)	25 (14.6)	
Parity			.56
0–4	277 (88.5)	36 (11.5)	
>4	136 (86.6)	21 (13.4)	
Timing of IUFD			.16
Antepartum	313 (86.7)	48 (13.3)	
Intrapartum	100 (91.7)	9 (8.3)	
HIV positive			.66
No	59 (89.4)	7 (10.6)	
Yes	350 (87.8)	50 (12.5)	
Birthweight (grams)			.004
Normal birthweight	213 (92.2)	18 (7.8)	
Low birthweight (<2500** **g)	199 (83.6)	39 (16.4)	
Baby sex			.48
Female	198 (89.2)	24 (10.8)	
Male	215 (87.0)	32 (13.0)	

Abbreviations: IUFD, intrauterine fetal death; MUAC, mid-upper arm circumference.

^*^Chi-square test.

Antimicrobial resistance was common in gram-negative organisms. Within the Enterobacterales group, 85% (n = 17) were resistant to ampicillin, and all were resistant to co-amoxiclav (n = 20). Sensitivity to amikacin was 95% (n = 19) compared with 35% (n = 7) for gentamicin, which may reflect prescribing practices within maternity services. No resistance to meropenem was detected (Supplementary Figure 1).

Group B streptococci were all sensitive to benzylpenicillin, with resistance more common in clindamycin (20%, n = 1) (Supplementary Figure 2). Within the viridans group, there was resistance to benzylpenicillin in just over 30% of isolates (n = 4), the majority of which were *Streptococcus anginosus* (n = 3) (Supplementary Figure 3). There were no cases of methicillin-resistant *S. aureus* within the stillbirth cohort (Supplementary Figure 4). Within the enterococci group, the majority of organisms were ampicillin sensitive (69%, n = 9), with 3 resistant organisms being *Enterococcus faecium* and the remainder an *Enterococcus* spp. that could not be identified further. There were no cases of vancomycin-resistant enterococci (Supplementary Figure 5). A full antibiogram of all isolated pathogens is available in Supplementary Figures 1–5.

## DISCUSSION

This study describes the infectious etiology of stillbirths in a large cohort of pregnant women in Kampala, Uganda, with a high proportion likely attributed to infection. Infection is an important cause of stillbirths globally, though limited data exist on the prevalence in low-resource settings and in Sub-Saharan Africa in particular [23, 24]. A 2018 systematic review highlighted infection as a leading cause of stillbirth in low-income countries (15.8%) [25]. However, data from low-resource settings are often limited by access to biological investigations. The ability of our study to characterize specific pathogens identified in heart blood aspirates from a large cohort of stillbirths provides vital data, which may inform therapeutic guidelines and safe maternity health care initiatives.

We report a culture rate of likely pathogens in 12.4% of cases, with *Enterococcus* spp. being the most isolated pathogen in our cohort. *Enterococcus faecalis* was reported as a common bacterium found in invasive fetal disease in a cohort of stillbirths from South Africa [26], and our data echo this finding, with *Enterococcus faecalis* being the most frequently isolated *Enterococcus* spp. Results from this study also highlighted GBS, *E. coli,* and *K. pneumoniae* as common pathogens, which mirrors our findings. Previous studies have reported GBS as a causative pathogen in 4% of stillbirths in Sub-Saharan Africa [27]. In our study, GBS was identified in <1% of stillbirths, though it remains among the most common pathogens isolated. Viridans streptococci have been isolated in cultures from stillbirths in high-resource settings [28] but, to our knowledge, has not been described previously within Africa. In an earlier paper in this series, we presented the results of neonatal sepsis surveillance in Uganda. Similar to these stillbirth results, *E. coli* was the most frequently isolated gram-negative bacterium, while *Streptococcus agalactiae*, viridans streptococci, and enterococci were the most frequently isolated gram-positive organisms [12].

In addition to the 12.4% of sampled stillbirths that had a pathogen identified from their blood culture, 6.6% of women were diagnosed with syphilis during pregnancy. We have previously reported the prevalence of syphilis infection in a birth cohort of women delivering from this site. The syphilis prevalence was much lower in the birth cohort compared with stillbirths, 61/3670 (1.7%, 1.3–2.1) vs 38/573 (6.6%, 4.7–9.0) [29]. This higher rate of syphilis among pregnant women delivering stillborn infants suggests that some of these deaths are likely due to infection. Syphilis has been demonstrated to be an important cause of stillbirth in low-income countries.

Our analysis highlights risks for infective etiology of stillbirth including low birthweight. It has been demonstrated previously that preterm labor and low-birthweight babies have increased risk of stillbirth [30, 31], and, in particular, infection as a cause of stillbirth [32–34]. A recent study using postmortem minimally invasive tissue sampling to investigate neonatal deaths and stillbirths in 7 low- and middle-income countries in Africa and South Asia found that around 60% of GBS-associated decedents were of LBW [35]. Our data also show that these stillborn infants are more likely to have other bacterial pathogens identified in blood culture. In the first paper in this supplement, we have demonstrated that preterm/low-birthweight infants also had increased odds of dying during their admission with suspected neonatal sepsis [12].

The antimicrobial resistance (AMR) data from this project, suggesting a high rate of resistance against amoxicillin, co-amoxiclav, and gentamicin within the Enterobacterales group, are concerning given the widespread use of beta-lactam antibiotics and aminoglycosides as first-line drugs for several maternal conditions [36]. AMR poses a significant risk to the health of pregnant women and their infants globally, with the risk felt disproportionately in low-resource settings. Gram-negative bacteria are a common cause of neonatal sepsis and are commonly resistant to first-line and second-line antimicrobials recommended by the World Health Organization (WHO) [37] and to carbapenems [38]. The BARNDARDS study flagged transmission of resistant bacteria between mother and child [39]. Our data highlight that these organisms are also likely a common infective cause of stillbirth, though we are unable to comment on preceding maternal colonization and nosocomial spread, which have also been identified as potential sources of infection for neonates [40, 41].

It was necessary to ship isolates to the UK for MALDI-TOF speciation as there was no access to identification of bacteria with mass spectrometry via MALDI-TOF in Uganda. This restricted our ability to differentiate between species of different bacteria (eg, enterococci) or to accurately speciate bacteria where basic bacteriology tests had failed (eg, streptococci species) at the study site. It is vital that efforts to strengthen laboratory science in low-resource settings are maintained, as capacity building is vital, not only for health system strengthening but also for grounding research within the setting from which data are being acquired.

### Limitations

The vast majority of stillbirths had heart blood aspirates collected (n = 569), however only 476 samples were analysed. This represents samples with insufficient volume for analysis and samples which were lost, either in transit or within facilities (n = 93, 16.7). This may introduce bias into our results.

Minimally invasive tissue sampling has been utilized in other studies to aid cause of death identification in stillbirths and neonates [42, 43] but was not performed in our study. Subsequently, the true rate of stillbirth with an infectious etiology may have been underascertained. A further limitation is that of comparison between those who consented to participate in this study and those who did not. We were able to recruit around one-third of women who had stillbirths over the course of our study. There may be important differences between these groups that we have not been able to consider in our analyses.

## CONCLUSIONS

Our study provides vital data highlighting the role of bacterial pathogens among a cohort of stillbirths in Kampala, Uganda. The high burden of stillbirths is a key driver of perinatal mortality and is a neglected global public health issue. The high proportion of stillbirths caused by likely pathogenic bacteria in this study highlights potential for prevention with prophylaxis and stresses the need for further research in this area.

## Supplementary Data

Supplementary materials are available at *Open Forum Infectious Diseases* online. Consisting of data provided by the authors to benefit the reader, the posted materials are not copyedited and are the sole responsibility of the authors, so questions or comments should be addressed to the corresponding author.

## Supplementary Material

ofae606_Supplementary_Data

## Notes

***Acknowledgments.*** We would like to thank the pediatricians at Kawempe National Referral Hospital, as well as the other staff and patients involved in the study. We would also like to thank all the staff at MUCML.

The complete PROGRESS Study Author Group is comprised of Abdelmajid Djennad, Agnes Nyamaizi, Agnes Ssali, Alexander Amone, Amusa Wamawobe, Annettee Nakimuli, Caitlin Farley, Carol Nanyunja, Christine Najuka, Cleophas Komugisha, Dan R. Shelley, Edward A. R. Portal, Ellie Duckworth, Emilie Karafillakis, Geraldine O’Hara, Godfrey Matovu, Hannah G. Davies, Janet Seeley, Joseph Peacock, Juliet Nsimire Sendagala, Katie Cowie, Kirsty Le Doare, Konstantinos Karampatsas, Lauren Hookham, Madeleine Cochet, Margaret Sewegaba, Mary Kyohere, Maxensia Owor, Melanie Etti, Merryn Voysey, Moses Musooko, Musa Sekikubo, Owen B. Spiller, Patience Atuhaire, Paul T. Heath, Philippa Musoke, Phiona Nalubega, Pooja Ravji, Richard Katungye, Ritah Namugumya, Rosalin Parks, Rose Azuba, Sam Kipyeko, Simon Beach, Stephen Bentley, Tim Old, Tobius Mutabazi, Valerie Tusubira, and Vicki Chalker.

***Author contributions.*** K.L.D., M.S., and P.M. conceived of the study design and methodology with input from A.W., O.B.S., and L.H. M.K., H.G.D., D.R., C.F., E.A.R.P., and J.P. were responsible for the conduct of the study and oversight of data collection activities. Data management activities were managed by V.T. A.W. was responsible for oversight of microbiology and optimization of the laboratory protocol, while L.H. was responsible for interpretation of microbiology results. K.L.D., M.K., H.G.D., L.H., V.T., D.R.S., C.F., E.A.R.P., S.B., M.S., O.B.S., and K.K. analyzed and interpreted the data. L.H., A.W., and V.T. wrote the first draft of this article. L.H., V.T., A.W., J.P., P.M., and K.L.D. drafted and edited the manuscript, and all authors reviewed and approved the final manuscript.

***Patient consent and ethics.*** Written informed consent was obtained from all patients involved in the study. This study was conducted in accordance with the principles set forth in the Harmonised Tripartite Guideline for Good Clinical Practice and the Declaration of Helsinki in its current version [44]. The study has been approved by the Ugandan National Council for Science and Technology (UNCST [45]; ref. HS 2496), Makerere University School of Medicine Research & Ethics Committee (SOMREC [46]; 2018–130), and St George's, University of London (SGUL REC [47] ref 2020.0024), which sponsored the study. The protocol was published in Gates Open Research in June 2020 [11].

***Data availability.*** The full data set for the PROGRESS study is available at doi:10.24376/rd.sgul.24101115.v1 [48].

***Financial support.*** This work was supported by the Bill and Melinda Gates Foundation (OPP1189053). V.T. also received funding from Makerere University – Johns Hopkins University Research Collaboration (MUJHU) to carry out this work. This work was also supported by the National Institute for Healthcare Research for the qualitative research (16/136/46). The content of the manuscript is solely the responsibility of the authors. The funders had no role in study design, data collection and analysis, decision to publish, or preparation of the manuscript.

***Supplement sponsorship***. This article appears as part of the supplement “Neonatal and Young Infant Infection in Uganda: the PROGRESS Study,” sponsored by St George's, University of London.
