# Supplementary material for: Infectious Causes of Stillbirths: A Descriptive Etiological Study in Uganda
Source: Open Forum Infect Dis. 2025 Mar 10;11(Suppl 3):S165–72. doi: 10.1093/ofid/ofae606 (PMC11891129; doi:10.1093/ofid/ofae606)
Supplement: ofae606_Supplementary_Data [file ofae606_supplementary_data.zip › Supplementary_Figures_4_and_5_Stillbirths.docx]

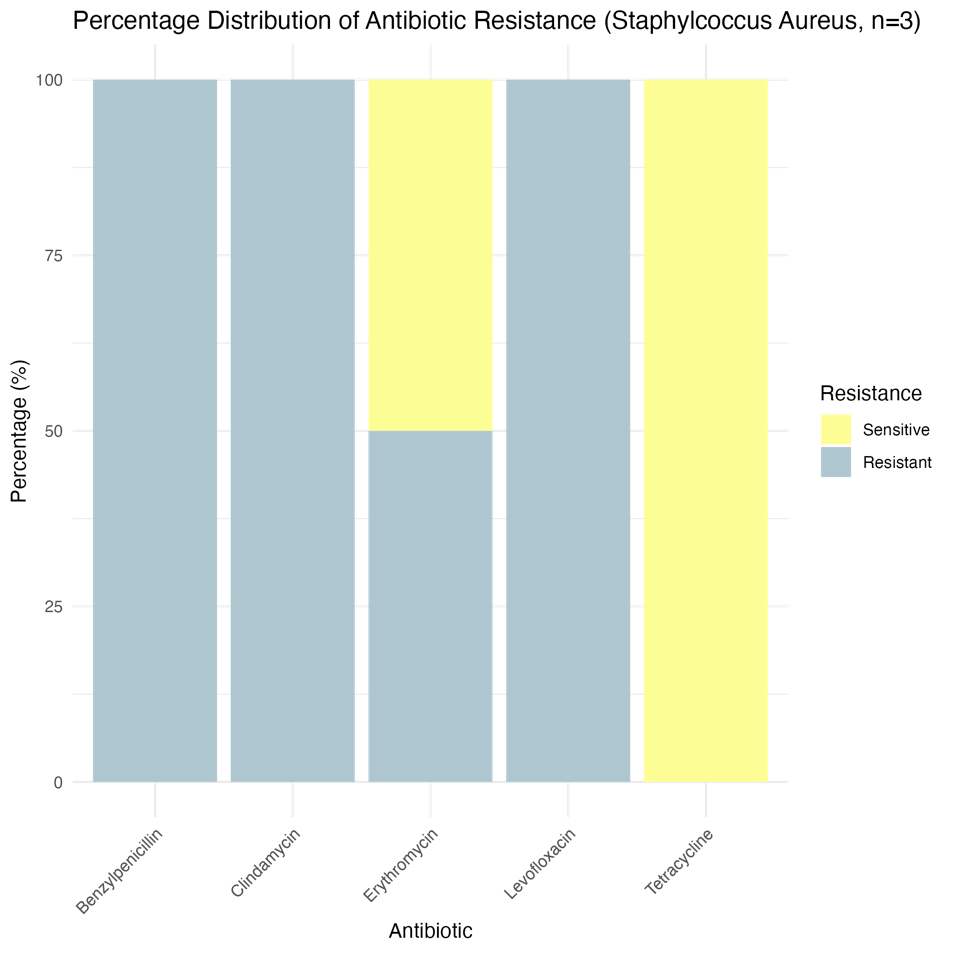


Supplementary Figure 4 Antibiogram for the staphylococcus aureus isolates.


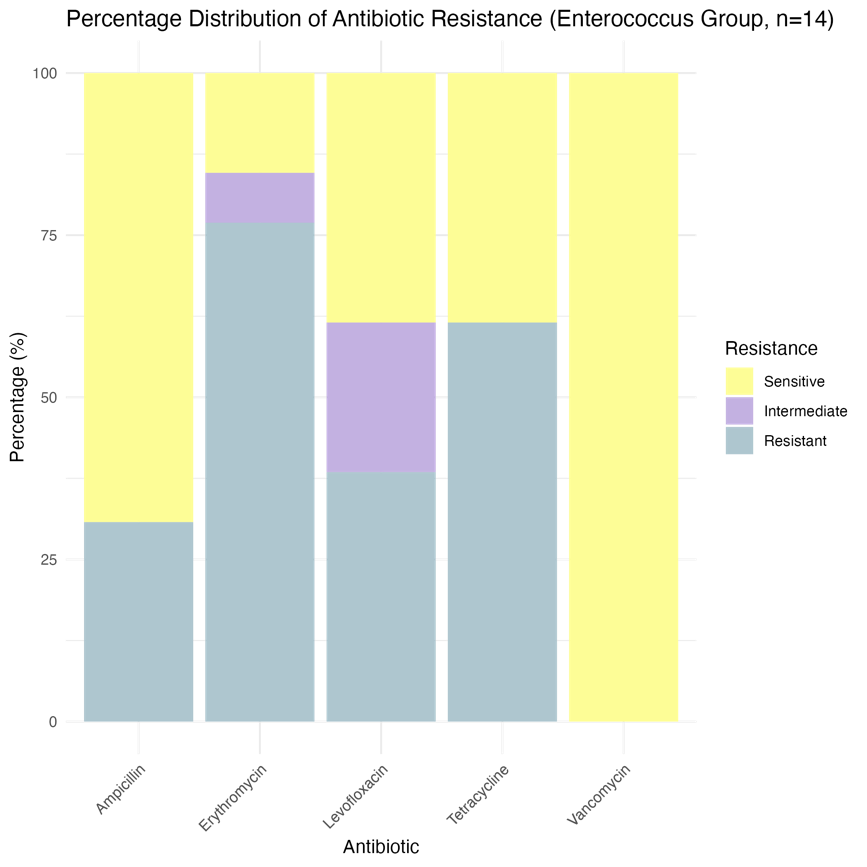


Supplementary Figure 5 - Antibiogram for the enterococci.
