# Supplementary material for: Infectious Causes of Stillbirths: A Descriptive Etiological Study in Uganda
Source: Open Forum Infect Dis. 2025 Mar 10;11(Suppl 3):S165–72. doi: 10.1093/ofid/ofae606 (PMC11891129; doi:10.1093/ofid/ofae606)
Supplement: ofae606_Supplementary_Data [file ofae606_supplementary_data.zip › Supplementary_Tables_Stillbirths.pdf]

Supplementary table 1 - Full list of organisms identified from 59 pathogenic blood cultures

Organism	Number	%
Acinetobacter colistinresistens	1	1.6
Enterobacter bugadensis	1	1.6
Enterococcus faecalis	9	14.3
Enterococcus faecium	5	7.9
Escherichia coli	13	20.6
Klebsiella pneumoniae	6	9.5
Leclercia adecarboxylata	1	1.6
Staphylococcus aureus	3	4.8
Stenotrophomonas maltophilia	1	1.6
Streptococcus agalactiae	5	7.9
Streptococcus anginosus	4	6.3
Streptococcus gallolyticus	1	1.6
Streptococcus infantarius	3	4.8
Streptococcus mitis oralis	5	7.9
Streptococcus salivarius	1	1.6
Viridans streptococcus	4	6.3
Total	63	100

*59 blood cultures were monomicrobial and 4 polymicrobial

Supplementary table 2 - Polymicrobial cultures with pathogens identified

Organism 1	Organism 2	Freq
Escherichia coli	Klebsiella pneumoniae	2
Klebsiella pneumoniae	Streptococcus anginosus	1
Escherichia coli	Streptococcus salivarius	1

Supplementary table 3 - Contaminants identified from 49 positive blood cultures

Contaminants	Freq	(%)
Bacillus	5	10.0
CoNS	21	42.0
Corynebacterium	7	14.0
Micrococcus	14	28.0
Rhodococcus	1	2.0
Weisella confusa	1	2.0
Lactobacillus jensenii	1	2.0
Total	50	100.0

*1 polymicrobial with >1 contaminant
