# Supplementary figures and images for: Infectious Causes of Stillbirths: A Descriptive Etiological Study in Uganda
Source: Open Forum Infect Dis. 2025 Mar 10;11(Suppl 3):S165–72. doi: 10.1093/ofid/ofae606 (PMC11891129; doi:10.1093/ofid/ofae606)

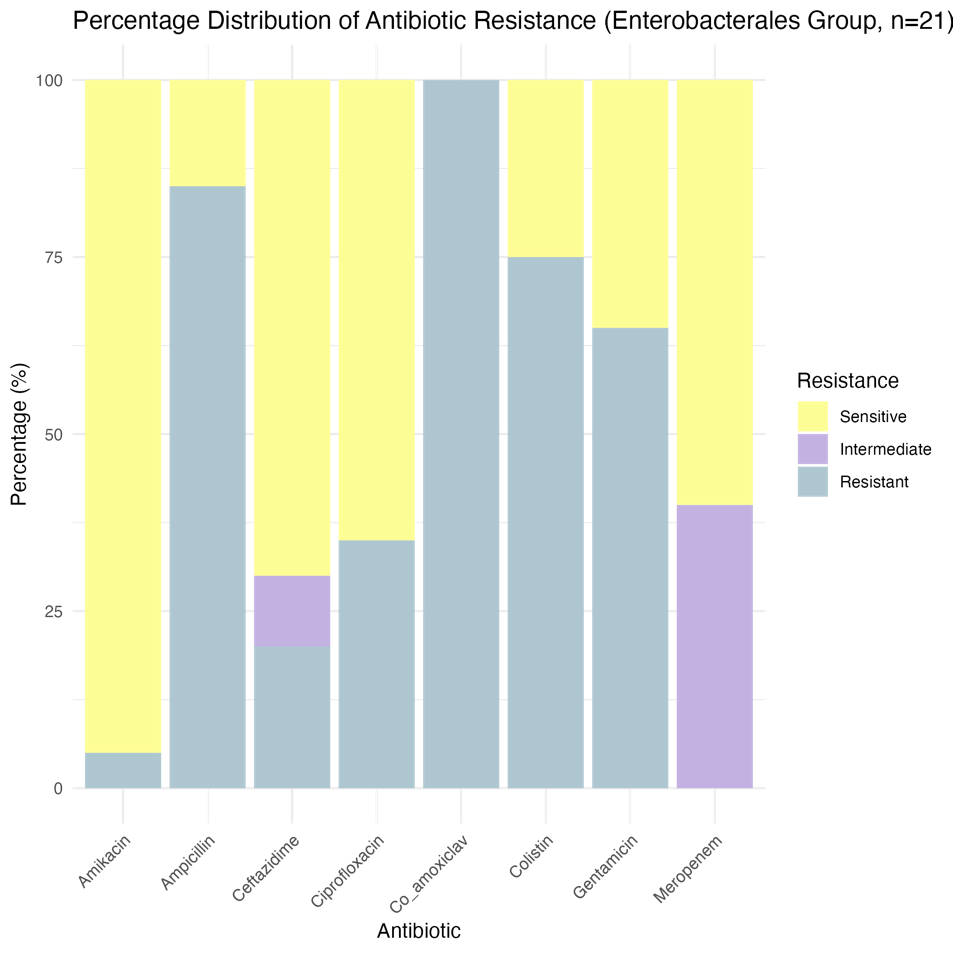


Supplementary Figure 1 - Antibiogram for the enterobacterales isolates.

Supplement: ofae606_Supplementary_Data [file ofae606_supplementary_data.zip › Supplementary_Figure_1_Stillbirths.docx]

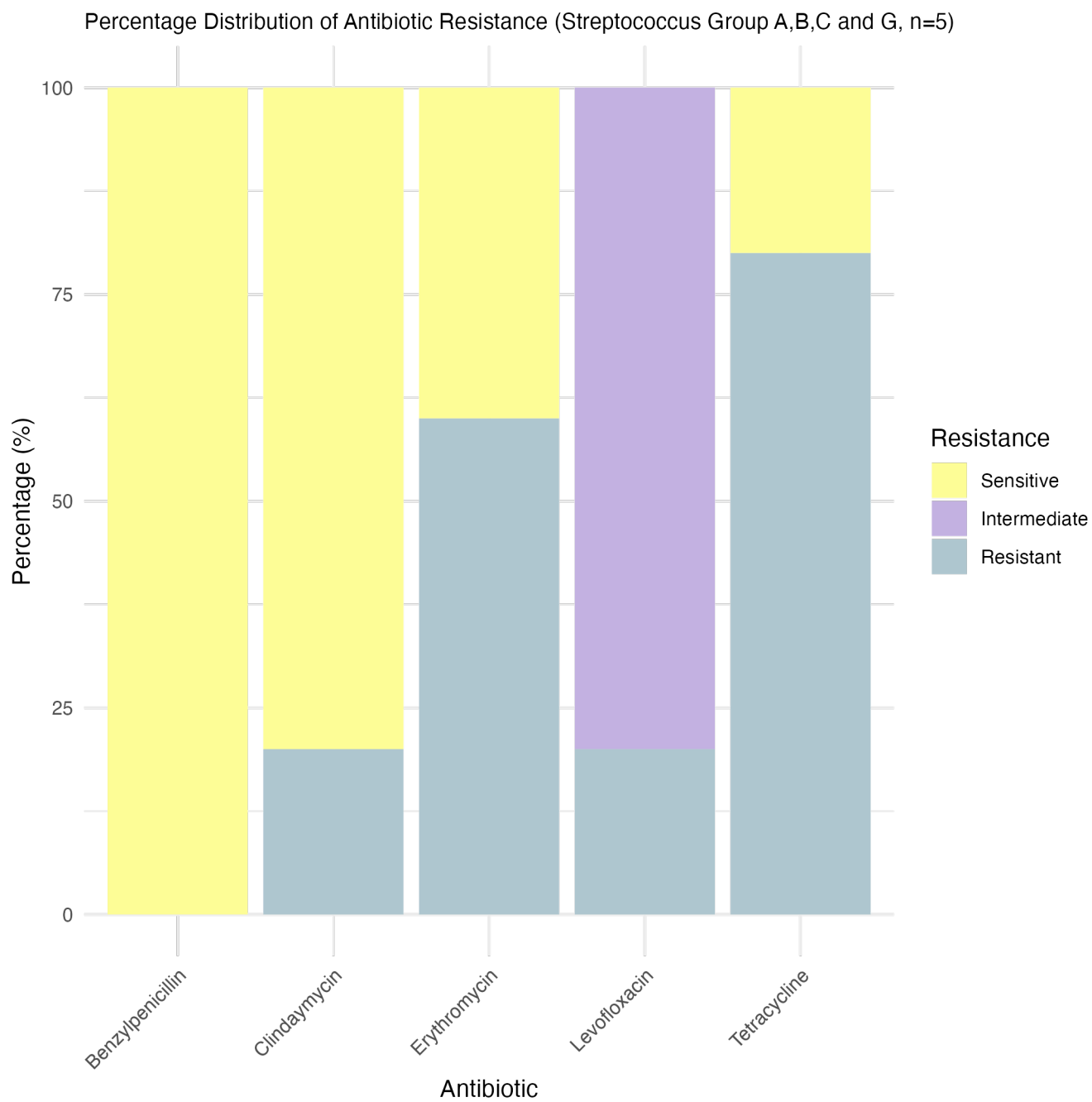

*Supplementary Figure 2 - Antibigram for streptococcal group A B and C isolates.*

Supplement: ofae606_Supplementary_Data [file ofae606_supplementary_data.zip › Supplementary_Figure_2_Stillbirths.pdf]

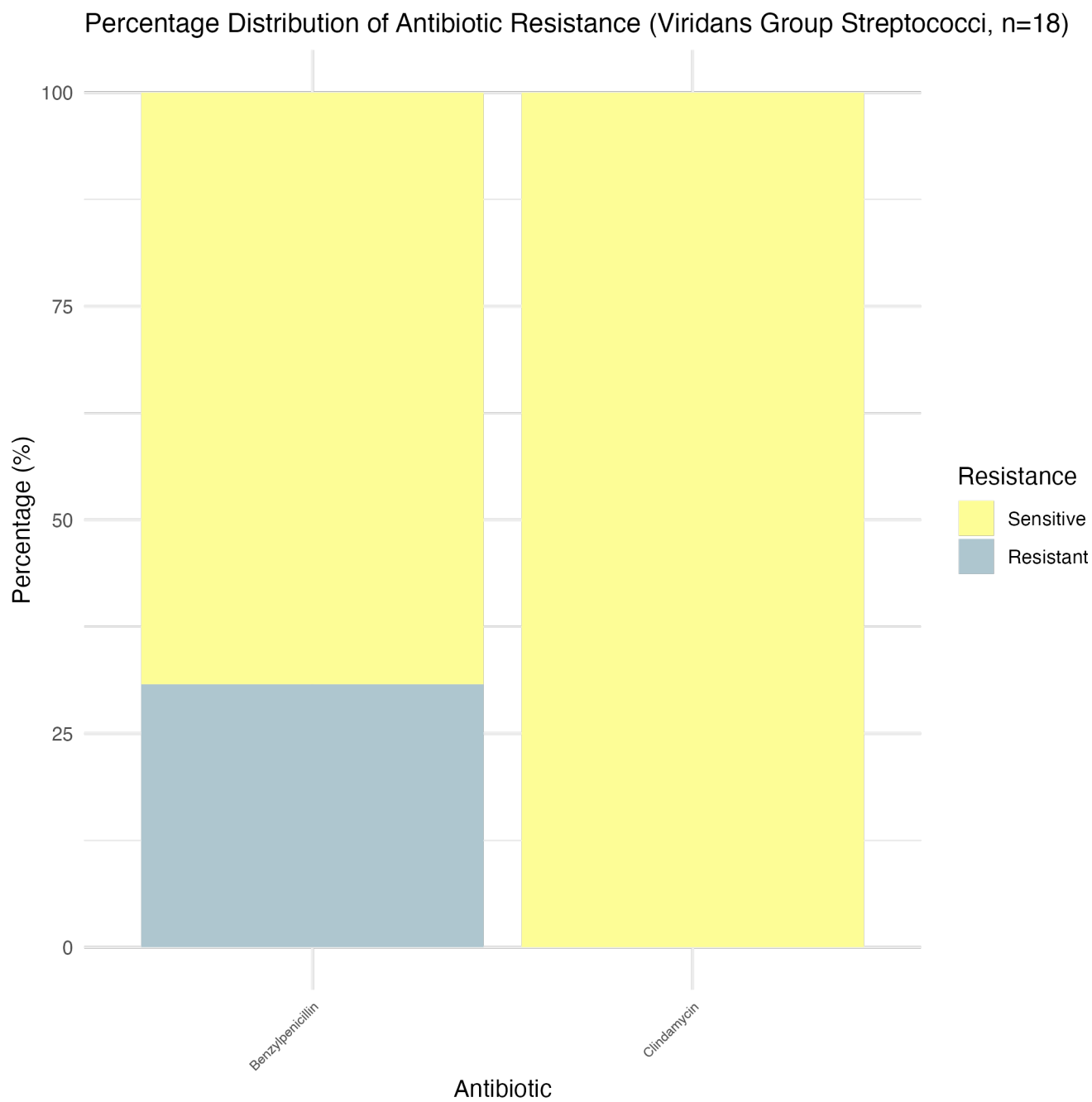

*Supplementary Figure 3 - Antibigram for the viridans group streptococci.*

Supplement: ofae606_Supplementary_Data [file ofae606_supplementary_data.zip › Supplementary_Figure_3_Stillbirths.pdf]
